# Association of skeletal muscle strength and cardiorespiratory fitness with bone mineral density: a cross-sectional study

**DOI:** 10.3389/fpubh.2025.1584610

**Published:** 2025-07-16

**Authors:** Beibei Wei, Zelong Miao, Xinge Yang, Shuning Chen, Xueying Guo, Jixiang Wang, Xu Huang, Hanping Zhang, Xuan Wang, Bin Jia

**Affiliations:** ^1^Project for Motor Function Evaluation and Scientific Training, Beijing DCN Orthopaedic Hospital, Beijing, China; ^2^School of Sport Science, Beijing Sports University, Beijing, China; ^3^Key Laboratory of Sports and Physical Health, Ministry of Education, Beijing, China

**Keywords:** isokinetic muscle strength test, cardiorespiratory endurance, bone density, QCT, Chinese adults

## Abstract

**Background:**

The high prevalence and increasing severity of osteoporosis have elevated it to a significant global public-health concern, imposing a substantial economic burden. Skeletal muscle strength and cardiorespiratory endurance serve as pivotal metrics in evaluating physical health. They play a vital role in mitigating the risks associated with bone density decline and the development of osteoporosis. This cross-sectional study was carried out among Chinese adults aged 30–60 years. Its aim is to investigate the associations between skeletal muscle strength, cardiorespiratory endurance, and bone density, thereby providing scientific evidence for formulating prevention and intervention strategies against osteoporosis.

**Method:**

A handgrip dynamometer was employed to measure the subjects’ grip strength, which served as an indicator for assessing their upper-limb muscle strength. Additionally, an isokinetic muscle-strength tester was utilized to determine the subjects’ lower–limb isokinetic muscle strength, thereby evaluating the strength of their lower–limb muscles. The exercise cardiopulmonary testing system was utilized to directly measure the subjects’ maximum oxygen uptake (VO₂ max) via a treadmill test. This crucial indicator was then employed to assess the subjects’ cardiorespiratory fitness (CRF). Moreover, the QCT bone density analysis system was used to measure the thoracolumbar bone density of the subjects, and their bone density levels were evaluated based on the *T* value. A multiple stepwise linear regression model was utilized to further examine the associations between the independent variables grip strength, quadriceps muscle strength, and maximum oxygen uptake and the dependent variable, the bone density *T* value, stratified by gender. A series of factors potentially influencing the results were adjusted for, such as age, weight, body mass index (BMI), smoking and drinking habits, as well as vitamin D and calcium levels.

**Results:**

In the final fully adjusted model, a significant positive correlation was detected between grip strength and the BMD T score (*β* = 0.03, *p* < 0.001). This correlation held significance in both women (*β* = 0.15, *p* < 0.001) and men (*β* = 0.07, *p* < 0.001). A significant correlation was observed between quadriceps muscle strength and the bone mineral density *T* score (*β* = 0.94, *p* < 0.001). Notably, this correlation was particularly pronounced in the female group, with a more significant relationship (*β* = 1.35, *p* < 0.001), whereas in the male group, the correlation was not significant (*β* = 0.42, *p* = 0.230). In addition, a significant correlation was identified between the maximum oxygen uptake and the bone density *T* value in the overall sample (*β* = 0.28, *p* = 0.009). Nevertheless, upon gender stratification, the correlation between the maximum oxygen uptake and bone density was not significant in women (*p* = 0.884), yet it was significant in men (*β* = 0.42, *p* = 0.009).

**Conclusion:**

In the 30–60 age group, a significant positive correlation was detected between skeletal muscle strength and bone density. Specifically, in women, lower limb muscle strength was more closely associated with bone density; however, this relationship was not significant in men. Moreover, the association between cardiorespiratory endurance and bone density varied by gender. It was not significant in women but demonstrated a significant positive correlation in the male group.

## Introduction

Osteoporosis is widely recognized as a serious public health problem (1). Osteoporosis increases the risk of falls and fractures, affects individual quality of life, and increases morbidity and mortality (2). A research has found that the prevalence of osteoporosis in developing countries is higher than that in developed countries (22.1% vs. 14.5%) (3). It is estimated that by 2035, the annual number of osteoporosis-related fractures in China will reach 4.83 million, causing annual losses of approximately US$19.92 billion (4).

Reduced bone density (BMD) will cause the fine structure of bones to be damaged and compressed to deform, easily causing osteoporosis. Research shows that spinal bone density usually reaches its peak at the age of 30 during human development, and then gradually decreases as age increases (5–7). However, osteoporosis caused by the bad living habits of modern people is not only seen in the elderly, but also in adults aged 30–60 years old (2). Current studies have confirmed that known risk factors for low bone density include advanced age, female gender, low body weight (3, 8, 9) as well as smoking, drinking (10), vitamin D and calcium deficiency (11). In addition, low grip strength and cardiorespiratory fitness (CRF) are very important risk factors (9, 12). Skeletal muscle has a significant impact on bone tissue formation. Muscle use and contraction impose loads on bones, causing them to adapt to these loads and thereby optimize their structure and strength (13). CRF, as an important indicator to measure the ability of the respiratory and cardiovascular systems to deliver oxygen to muscles during exercise, is not only an objective reflection of an individual’s habitual physical activity, but also a powerful proof of an individual’s healthy life trajectory (14–16). Therefore, skeletal muscle strength and cardiorespiratory endurance, as key indicators of physical health (17), are crucial to reducing bone density and the risk of osteoporosis.

Previous studies on the relationship between skeletal muscle and bone density indicate that upper limb grip strength shows a stronger correlation with bone density (9, 18), particularly in females compared to males (19). However, relying solely on upper limb grip strength as a predictor of overall skeletal muscle strength may not be sufficient (20), as lower limb muscle strength could have a more direct association with bone health (18). Despite this, the specific influence of lower limb skeletal muscle strength on bone density is still unclear. Similarly, research on CRF and bone density suggests that higher levels of CRF were associated with a reduced risk of osteopenia. Most studies assess CRF indirectly through estimated maximal oxygen uptake, a practical but less precise method. The gold standard for CRF evaluation—direct measurement of oxygen uptake using gas analysis during graded exercise testing—is more accurate but also more resource-intensive (17). Currently, there is limited evidence on the use of CRF as a prognostic tool for osteopenia and osteoporosis risk in Chinese adults.

This multidisciplinary study, conducted through collaboration between orthopedic physicians, rehabilitation specialists, and sports science researchers at a leading Chinese orthopedic hospital, represents a significant advancement in musculoskeletal research. By employing an innovative integration of gold-standard CPET protocols, high-resolution dynamometry, and standardized bone density measurements, we provide the first comprehensive characterization of the muscle strength-CRF-bone density relationship in Chinese adults. Our approach overcomes key limitations of previous studies by replacing subjective assessments with objective, quantitative measures, yielding unprecedented precision in physiological evaluation. The novel assessment framework developed through this research offers clinically actionable insights for osteoporosis risk stratification in this population. Importantly, our identification of modifiable predictors establishes an evidence base for developing targeted exercise regimens and personalized prevention strategies, bridging the gap between methodological innovation and practical clinical application in osteoporosis management.

## Materials and methods

### Participant characteristics

From October 2023 to January 2024, Beijing Delconi Orthopedic Hospital recruited external subjects. Subjects are required to be ≥30 years old and ≤60 years old, regardless of gender, be able to carry out normal activities, have sound development, and have no symptoms. Obvious physical defects, able to correctly understand test requirements and cooperate to complete the test. People with pre-existing metabolic bone disease or taking any medications that may affect bone density were excluded from enrollment. All subjects gave written informed consent and were informed of study details before participating in the study. Subjects were required to fill out self-report questionnaires regarding overall physical health, bad lifestyle habits (smoking, drinking) and other information, and completed medical health examinations, skeletal muscle strength tests, cardiorespiratory endurance tests and bone density assessments. This research was approved by the Ethics Committee of Beijing Delconi Orthopedic Hospital. This research was supported by the project fund as a sub-project of the Science and Technology Innovation Project of the State Sports General Administration of China.

### Medical checkup

The subjects underwent extensive medical health examinations at the hospital, including height, weight, waist circumference, body composition analysis, and blood tests. Height and weight (unit: cm, kg) were measured using a height and weight scale. The subjects were asked to take off their shoes, wear single clothes and pants, keep their heels together and make an angle of 60° between their toes, look straight ahead with both eyes, and stand at attention. Body mass index (BMI) is weight/height^2^ (unit: kg/m^2^). Use a soft tape to measure waist circumference (unit: cm). The soft tape measures the horizontal circumference through the center of the umbilicus or the midpoint line between the lowest point of the ribs and the upper edge of the iliac crest. This process does not put any pressure on the skin. Body composition was measured using a body composition analyzer (X-Scan Plus II, JAWON, Korea). The subjects were required to fast for 12 h and then blood samples were collected from the antecubital vein after arriving at the hospital. In this study, 25(OH) D and serum calcium ion levels were selected as bone metabolism indicators, with reference value ranges of 30–100 ng/ml and 1.42–1.90 mmol/L, respectively.

### Isokinetic muscle strength test

The maximum muscle strength of quadriceps muscles in both knee joints was tested using the multi-joint isokinetic muscle strength Test and Rehabilitation Training System (HUMAC NORM, CSMI, USA). Isokinetic muscle strength test is recognized as a reliable and effective muscle strength test and is used as a reference for other muscle tests (21). Peak moment is the expression of the maximum moment during joint flexion and extension and the expression of the maximum muscle strength of the lower extremity, and is regarded as the gold standard in isokinetic testing. The absolute peak moment of the quadriceps muscle was chosen as the maximum muscle force of the lower extremity (N·m/kg) because of the difference in body weight of the subjects.

Before the test, the subjects warmed up on a stationary bicycle for 10 min (60–70 revolutions per minute). After that, the subjects kept upright in the seat, the axis of the knee joint was kept in a straight line with the rotation center of the power head, and the test Angle was set at 0°-90°, 0° meant that the knee joint was extended 0 degrees, and the resistance pad at the end of the power arm was fixed at 3 cm from the upper edge of the ankle joint. In order to reduce compensation during the test, the torso and test legs are held in place using a holding band. In order to get familiar with the test system, before the formal test, the subjects first performed 4 attempts of isokinetic flexion and extension with an angular velocity of 60°/s, and then completed 5 formal knee flexion and extension exercises. After the left and right legs were tested separately, the maximum value was taken as the maximum muscle strength of the lower limbs.

### Hand grip measurement

Grip strength testing is a practical and effective method to measure muscle strength, which has been clinically proven to be an indicator of adverse health outcomes (22, 23). Grip strength testing is used to assess upper limb muscle strength. The subjects were measured using an electronic handgrip dynamometer (EH101, CAMRY, China). The subjects were required to squeeze the handgrip dynamometer with one hand with the greatest force, and repeat the above operation twice with both hands. The maximum value was taken as the maximum muscle strength of the upper limb.

### CPET for VO_2max_

The subjects underwent an incremental load cardiopulmonary endurance test using the Bruce treadmill mode on an exercise cardiopulmonary testing system (Master Screen CPX, Jaeger, Germany) (24). The amount of gas inhaled and exhaled during exercise, including oxygen and carbon dioxide, was measured, and the maximum oxygen uptake (VO_2max_) was used to evaluate cardiopulmonary endurance (25).

The subjects started with a warm-up walk and entered the exercise phase after a 3-min warm-up. Starting from the first phase, the treadmill automatically entered the next phase every 3 min. There were 7 exercise phases in total, and each phase corresponded to different speeds and slopes, as shown in Table 1. During the entire exercise process, blood pressure, blood oxygen saturation and electrocardiogram were monitored in real time. The subjects wore a Polar heart rate belt (Polar H9, USA) to record the exercise heart rate in real time, and the Rating of Perceived Exertion (RPE) scale was used to evaluate the level of exercise fatigue (26). When the following two conditions occurred at the same time, the subjects could stop exercising, and the heart rate measured immediately was HRmax (27): (1) When the exercise load continued to increase according to the Bruce protocol, the real-time heart rate no longer increased; (2) When the exercise load continued to increase, the real-time VO_2max_ no longer increased; (3) When the subject subjectively felt that he could not maintain the exercise, the test should be terminated; (4) When the respiratory quotient (RER) ≥ 1.05. In the Ergospirometry software, VO_2max_ refers to the average value of VO_2_ (in liters/min) measured by 8 consecutive breaths during the peak exercise test. In order to ensure that the VO_2max_ data of each subject is accurate, the data needs to be manually selected to verify that the selected VO_2max_ value is correct. Lastly, all VO₂ max values were weight-standardized (unit: mL/min/kg) to control for the confounding effects of body weight variation across participants.

**Table 1:** Improved Bruce scheme.

Rank	Speed (mp/h)	Speed (km/h)	Slope (%)	METs	Time (min)
1	1.7	2.7	10	4	3
2	2.5	4.0	12	7	6
3	3.4	5.4	14	9	9
4	4.2	6.8	16	13	12
5	5.0	8.0	18	16	15
6	5.5	8.8	20	19	18
7	6.0	9.6	22	22	21

### Bone mineral density (BMD)

According to the World Health Organization (WHO), the diagnosis of bone disease is mainly based on bone density measurements at the femoral neck and lumbar spine, where fractures occur most frequently and are associated with higher morbidity and mortality (28). In this study, we used the QCT bone density measurement system (Mindways Image QCT, Mindway, USA) to accurately assess the subjects’ bone density. QCT technology, as a three-dimensional imaging technology, uses multi-slice spiral CT scanning to accurately quantify BMD and can distinguish between cancellous bone and cortical bone. It has high sensitivity for detecting bone mineral loss in the thoracic and lumbar spine (29). In contrast, conventional dual-energy X-ray absorptiometry (DXA), although widely used clinically to measure BMD at the lumbar spine and proximal femur, cannot distinguish between cancellous bone and cortical bone. While DXA remains the gold standard for low-risk, high-population screening, QCT provides superior structural data at the cost of higher radiation exposure. Therefore, the prevalence of osteoporosis detected by DXA is generally lower than that detected by QCT technology (30).

In this study, bone density tests were performed by experienced CT doctors, who accurately scanned the subjects’ thoracic spine (T11-T12) and lumbar spine (L1-L4) to calculate the average bone density (BMD, in g/cm^3^) and T value of the area.

### Statistical analyses

All data are expressed as mean ± standard deviation (x ± s), and SPSS26.0, R4.2 and Origin2024 were used for data analysis and chart drawing. Independent sample T test was used to analyze the significant differences in results between genders. The linear relationship between the key variables grip strength, quadriceps strength, VO_2max_ and the outcome variable (*T* score) was described by calculating the Pearson correlation coefficient (*r*) and drawing a scatter plot. To further explore the effects of skeletal muscle strength and cardiorespiratory endurance on bone mineral density, a multiple stepwise linear regression model was used to gradually adjust possible confounding factors for stratified analysis. The statistical significance level was set at *α* = 0.05. Significance was indicated as *p* < 0.05, very significant as *p* < 0.01, and extremely significant as *p* < 0.001.

## Results

### Participant characteristics

A total of 231 subjects were recruited for this study, and 176 subjects finally met the inclusion criteria and participated in the complete test, including 90 males and 86 females, with a participation rate of 76.2%. The average age of the subjects was 44.5 ± 8.0 years old. The results of the subjects’ age, weight, height, BMI, waist circumference, bone density T value, grip strength, VO_2max_, quadriceps strength, 25(OH)D, and Ca^2+^ are shown in Table 2. The results of the independent sample T test showed that there was no statistical difference in age between male and female subjects, but there were significant differences in weight, height, BMI, waist circumference, grip strength, VO_2max_ and quadriceps strength between males and females (*p* < 0.01).

**Table 2:** Descriptive characteristics.

Characteristics	All(176)	Women(86)	Men(90)	*p*
Age(years)	44.5 ± 8.0	43.9 ± 8.0	45.2 ± 8.1	0.28
Height(cm)	167.9 ± 8.5	161.1 ± 4.7	174.4 ± 5.7	**<0.001**
Weight(kg)	68.9 ± 12.1	59.5 ± 6.3	77.9 ± 9.0	**<0.001**
BMI (kg/m^2^)	24.3 ± 2.7	22.9 ± 2.5	25.6 ± 2.3	**<0.001**
Waist Circumference(cm)	85.7 ± 10.6	79.1 ± 8.7	92.0 ± 8.1	**<0.001**
T score	−0.20 ± 1.16	−0.33 ± 1.17	−0.08 ± 1.14	0.15
Hand Grip(kg)	37.88 ± 10.85	28.24 ± 3.52	47.09 ± 6.67	**<0.001**
VO_2max_(mL/min/kg)	**39.42 ± 8.17**	**42.23 ± 8.59**	**36.49 ± 6.57**	**<0.001**
Quadriceps strength(N·m/kg)	1.48 ± 0.37	1.30 ± 0.35	1.66 ± 0.29	**<0.001**
25(OH)D(ng/mL)	27.71 ± 7.69	28.17 ± 8.78	27.26 ± 6.49	0.43
Ca^2+^(mmol/L)	1.50 ± 0.04	1.50 ± 0.05	1.49 ± 0.04	0.05

All characteristics reported as mean ± SD, where SD represents Standard Deviation. BMI, Body Mass Index; VO_₂max_, Maximal Oxygen Uptake; 25 (OH) D, 25 - Hydroxyvitamin D; Ca^2+^, Calcium ion. Values with *p* < 0.001 indicate extremely significant differences between men and women groups; *p* < 0.01 indicates significant differences.

### Bivariate correlations of all indicators

**Figure 1:**
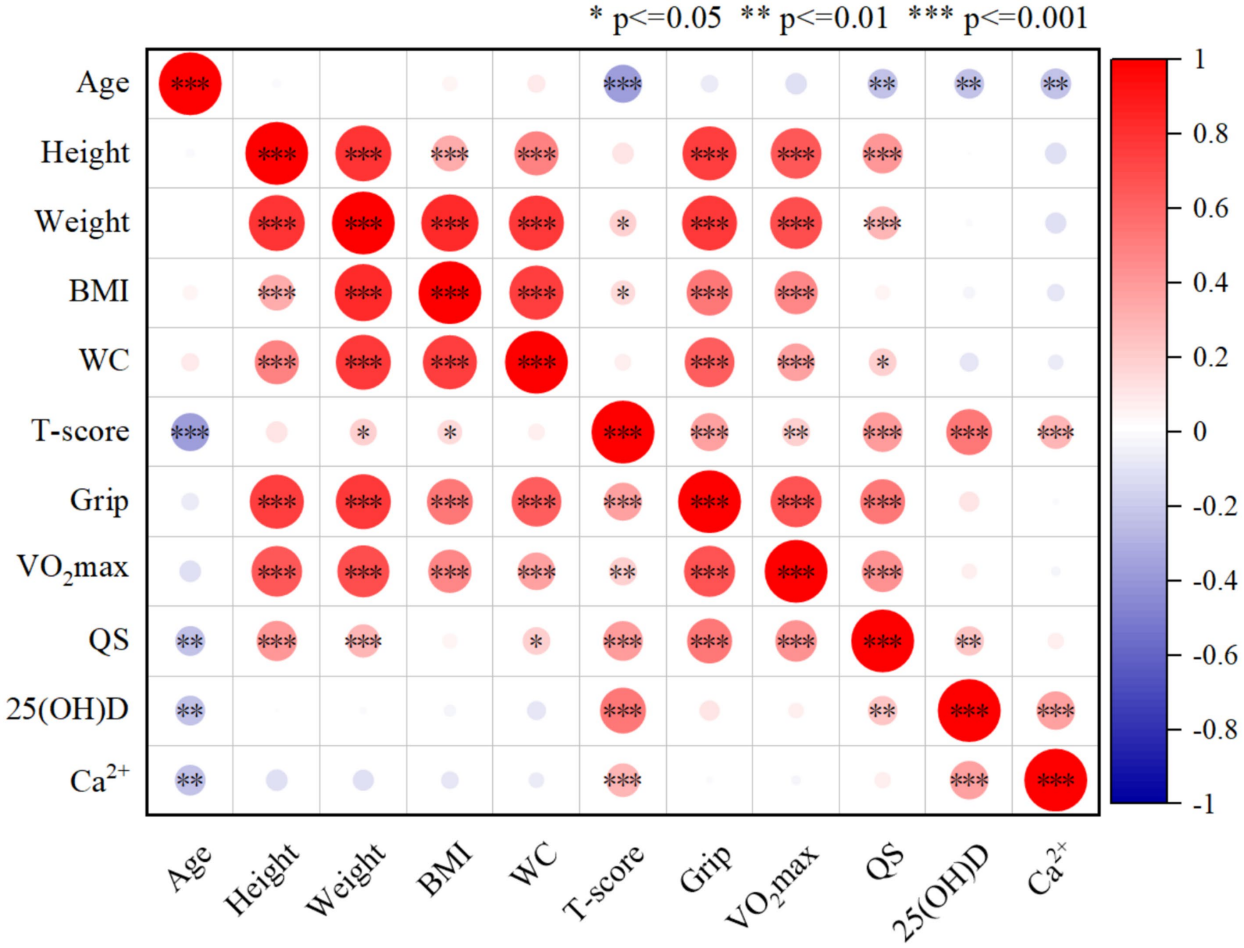
The correlation analysis between two indicators (*n* = 176). Significant associations (*p* < = 0.05) of the comparison between each index are indicated by *.

As shown in Figure 1, there was a significant positive correlation between bone density *T* and grip strength (*r* = 0.364, *p* < 0.001), quadriceps strength (*r* = 0.398, *p* < 0.001) and VO_2max_ (*r* = 0.664, *p* = 0.009). There was a significant positive correlation between 25(OH)D content (*r* = 0.528, *p* < 0.001) and Ca^2+^ content (*r* = 0.288, *p* < 0.001) and bone density *T* value. Older age was significantly correlated with lower bone density T (*r* = −0.365, *p* < 0.001); there was a weak positive correlation between weight (*r* = 0.184, *p* < 0.05) and BMI (*r* = 0.151, *p* < 0.05) and bone density *T*.

### Associations of skeletal muscle strength and BMD

**Figure 2:**
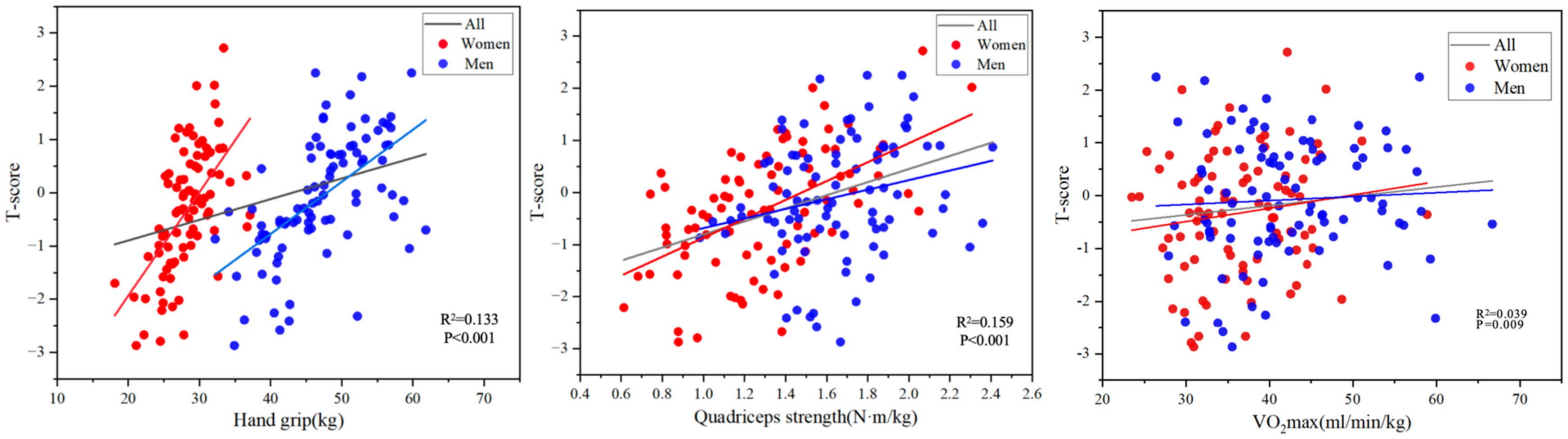
The correlations between Hand grip, Quadriceps strength and VO2max vs. BMD at the spine and hip (*n* = 176).

In the linear regression model, greater grip strength was significantly associated with higher *T* scores, even after adjustment for confounding variables (Table 3 and Figure 2, *R*^2^ = 0.133, *p* < 0.001). After stratifying the indicators by gender and adjusting for covariates, there was a significant positive correlation between women’s grip strength and *T*-score (*p* < 0.001). After adjustment for Model 3 covariates, there was no significant correlation between grip strength and *T*-score in men (*p* = 0.355).

**Table 3:** Differences in BMD *T*-score at the thoracolumbar spine (T11-T12, L1-L4) per unit, hand grip and quadriceps strength.

Variables	Group	Model 1			Model 2			Model 3			Model 4		
		*β* (95%CI)	*p*	*R* ^2^	*β* (95%CI)	*p*	*R* ^2^	*β* (95%CI)	*p*	*R* ^2^	*β* (95%CI)	*p*	*R* ^2^
Hand grip	Women (86)	0.19 (0.14 ~ 0.25)	**<0.001**	**0.33**	0.17 (0.11 ~ 0.22)	**<0.001**	**0.41**	0.24 (0.07 ~ 0.41)	**0.013**	**0.02**	0.15 (0.09 ~ 0.21)	**<0.001**	**0.33**
	Men (90)	0.10 (0.07 ~ 0.13)	**<0.001**	**0.32**	0.09 (0.06 ~ 0.12)	**<0.001**	**0.33**	0.05 (−0.05 ~ 0.15)	**0.355**	**0.51**	0.07 (0.05 ~ 0.10)	**<0.001**	**0.35**
	All (176)	0.04 (0.02 ~ 0.05)	**<0.001**	**0.13**	0.04 (0.02 ~ 0.05)	**<0.001**	**0.24**	0.09 (0.03 ~ 0.16)	**0.010**	**0.18**	0.03 (0.02 ~ 0.05)	**<0.001**	**0.30**
Quadriceps strength	Women (86)	1.81 (1.21 ~ 2.42)	**<0.001**	**0.28**	1.52 (0.93 ~ 2.11)	**<0.001**	**0.37**	2.91 (0.88 ~ 4.95)	**0.013**	**0.17**	1.35 (0.77 ~ 1.94)	**<0.001**	**0.35**
	Men (90)	0.92 (0.13 ~ 1.71)	**0.024**	**0.05**	0.56 (−0.25 ~ 1.38)	**0.180**	**0.10**	1.11 (−0.74 ~ 2.96)	**0.271**	**0.48**	0.42 (−0.26 ~ 1.10)	**0.230**	**0.32**
	All (176)	1.26 (0.83 ~ 1.69)	**<0.001**	**0.16**	1.05 (0.63 ~ 1.47)	**<0.001**	**0.23**	1.87 (0.64 ~ 3.11)	**<0.001**	**0.21**	0.94 (0.56 ~ 1.32)	**<0.001**	**0.23**

Model1: Unadjusted covariates. Model2: Adjusted age. Model3: Weight, BMI, smoking (only in men), alcohol consumption were further adjusted based on the previous model. Model4: Further adjustments were made for, 25(OH)D, Ca^2+^ . P value indicates statistical significance (*p* < 0.001 indicates that the grip strength/quadriceps strength is extremely significant with the BMD T score, *p* < 0.01 indicates that the statistical result of the relationship between grip strength/quadriceps strength and BMD T score is significant. R^²^ (coefficient of determination) represents the proportion of variance in the BMD T - score explained by the model. Higher R^²^ values suggest greater explanatory power of the model for the outcome variable.

In the unadjusted model, greater quadriceps strength was significantly associated with greater *T* in all subjects and after adjustment for confounding variables (Table 3 and Figure 2, *R*^2^ = 0.159, *p* < 0.001). After stratifying the indicators by sex and adjusting for covariates, quadriceps muscle strength in women was significantly positively correlated with *T*-score (*p* < 0.001). In the unadjusted model, there was a significant positive correlation between male quadriceps muscle strength and *T*-score (*p* = 0.024), but after adjustment for covariates in Model 2, Model 3, and Model 4, there was no significant correlation between male quadriceps muscle strength and *T*-score (*p* = 0.180).

### Associations of VO_2max_ and BMD

A greater VO_2max_ was significantly correlated with a greater *T* value in all subjects (Figure 2, *R*^2^ = 0.039, *p* = 0.009). After adjusting the covariate age in model 2, VO_2max_ of all subjects were significantly correlated with *T* value (of 0.22, *p* = 0.028), but after adjusting the covariate weight in model 3, the correlation between VO_2max_ and *T* value of subjects was not significant (*p* = 0.264). After adjusting BMI, smoking, drinking, 25(OH)D, and Ca^2+^ in models 4 and 5, the subjects’ VO_2max_ was significantly correlated with T value (*p* = 0.024, *p* = 0.008).

After stratifying the indicators by gender and adjusting for covariates, there was no significant correlation between VO_2max_ and *T* value in women. In the unadjusted linear regression model, there was a significant positive correlation between VO_2max_ and *T* value in men (*p* = 0.009). After adjustment for covariates, the correlation between VO_2max_ and *T* value in men was not significant Table 4.

**Table 4:** Differences in BMD T-score at the thoracolumbar spine (T11–T12, L1–L4) per unit and VO_2max_.

Variables	Group	Model1			Model2			Model3			Model4			Model5		
		*β* (95%CI)	*p*	*R* ^2^	*β* (95%CI)	*p*	*R* ^2^	*β* (95%CI)	*p*	*R* ^2^	*β* (95%CI)	*p*	*R* ^2^	*β* (95%CI)	*p*	*R* ^2^
VO2max	Women	−0.05 (−0.74 ~ 0.64)	**0.884**	**0.01**	−0.24 (−0.86 ~ 0.38)	**0.453**	**0.18**	0.35 (−1.22 ~ 1.92)	**0.668**	**0.03**	0.05 (−0.62 ~ 0.73)	**0.875**	**0.18**	0.12 (−0.46 ~ 0.71)	**0.679**	**0.04**
	Men	0.42 (0.11 ~ 0.73)	**0.009**	**0.06**	0.30 (−0.02 ~ 0.62)	**0.069**	**0.11**	0.21 (−0.66 ~ 1.09)	**0.640**	**0.05**	0.21 (−0.07 ~ 0.49)	**0.143**	**0.46**	0.11 (−0.17 ~ 0.40)	**0.431**	**0.32**
	All	0.28 (0.07 ~ 0.49)	**0.009**	**0.03**	0.22 (0.03 ~ 0.42)	**0.028**	**0.15**	0.44 (−0.32 ~ 1.21)	**0.264**	**0.20**	0.28 (0.04 ~ 0.53)	**0.024**	**0.28**	0.24 (0.06 ~ 0.42)	**0.008**	**0.14**

Model1: Unadjusted covariates. Model2: Adjusted age. Model3: Adjusted Weight. Model4: BMI, smoking, alcohol consumption were further adjusted based on the previous model. Model5: Further adjustments were made for 25(OH)D, Ca^2+^. P value indicates statistical significance (*p* < 0.001 indicates that VO_2max_ is extremely significant with the BMD T score, *p* < 0.01 indicates that the statistical result of the relationship between VO_2max_ and BMD T score is significant. R^²^ (coefficient of determination) represents the proportion of variance in the BMD T - score explained by the model. Higher R^²^ values suggest greater explanatory power of the model for the outcome variable.

## Discussion

In adults aged 30–60 years, greater grip strength and quadriceps strength were associated with higher bone mineral density. When further analyzing the relationship between quadriceps muscle strength and bone density, in men, after adjusting for all confounding factors, the correlation between quadriceps muscle strength and bone density was no longer significant; In women, the relationship between quadriceps strength and BMD was not affected by these adjustments, suggesting that women may exhibit different biological or physiological mechanisms for this relationship. Gender plays an important role in the relationship between muscle strength and bone density.

Studies have shown that cardiorespiratory endurance is significantly correlated with bone density. In women, there was no significant correlation between cardiorespiratory endurance and bone density, but in men, there was a significant correlation between cardiorespiratory endurance and bone density.

There was a significant positive correlation between grip strength and quadriceps strength and bone mineral density, supporting skeletal muscle strength as an important indicator of bone health. The association was stronger in women than in men. This is consistent with findings from India and Gambia, where women had a stronger association between muscle strength and bone density than men (19, 31). According to Wolff’s law, bones are able to adapt to external stresses caused by muscle forces and reshape accordingly. This adaptability suggests that skeletal muscle strength is a key external factor affecting bone density and contributes to adaptive bone remodeling. There are gender differences in hormone levels, genetic factors, and exercise habits, which may make the female skeletal system physiologically more effective in responding to changes in muscle strength, and this adaptability can be used to improve bone health.

This study adds to the evidence that lower extremity quadriceps strength is associated with BMD. Unlike previous studies that focused on indirect indicators such as gait speed, the stand-and-go test, and the five-sit-up test (17), this study used isokinetic muscle strength testing to directly measure the peak torque of the quadriceps femoris, which is considered to be the golden indicator for assessing muscle strength. This study is consistent with the results of Chen F et al. (32), which showed that the 1RM (maximum number of repetitions per squat) of Chinese adult males was positively correlated with local and total bone density. The study by Huawei Han et al. also showed that quadriceps muscle mass and strength were significantly positively correlated with bone density (33). In addition, Misch et al. found that knee and ankle extensor strength was significantly positively correlated with bone density in 60-year-old women. This study further extended these findings, indicating that decreased quadriceps strength can serve as a predictor of decreased bone density in adults aged 30–60 years. Further design of quadriceps muscle strength exercise intervention programs is needed to explore effective strategies for preventing bone mineral density loss.

This study confirmed a significant positive correlation between cardiorespiratory fitness (CRF) and bone mineral density (BMD). As an important indicator of physical activity levels, CRF can improve bone health by promoting bone cell activation and bone resorption/formation homeostasis through the mechanical load generated by exercise (34). This finding is consistent with the findings of Wainstein et al. (35) in a study of 2,569 men aged 50–90 years, which confirmed that high CRF reduces the risk of BMD loss and osteoporosis in the femoral neck. Schwarz et al. (18) further supported this association in Danish men aged 31–60 years, showing that CRF was positively correlated with the total coxal-lumbar BMD t score even after adjusting for confounding factors such as age, body weight, and smoking. Gouveia et al. (36) reached a similar conclusion in a cohort study of 802 older Portuguese adults. Combined with available evidence, high CRF has a positive effect on maintaining BMD levels and reducing the risk of osteoporosis.

In women, the association between CRF and BMD was not statistically significant. Biological mechanisms show that estrogen, as a key regulator of bone metabolism, affects bone mineral density by regulating receptor activity and osteoblast function (37). Due to the high sensitivity of bone to hormonal changes, the sudden decline of estrogen during menopause may amplify its effect on bone metabolism in women (38), which may explain the masking of CRF effects by endocrine factors (39). It is worth noting that studies have found a significant correlation between CRF and BMD in postmenopausal women (12), which is in contrast to the results of this study. This difference may be due to the study design: This study included a cross-age population of 30–60 years old, and the heterogeneity of bone metabolic status in postmenopausal women may weaken the overall association. However, the protective effect observed in the male study (35) was also not significant after adjusting for covariates such as age and BMI in this sample, which may be related to the decrease in statistical efficacy caused by sample size reduction.

In summary, after controlling for age, body composition, lifestyle and nutritional factors, there is a significant association between CRF and BMD in adults aged 30–60 years, but there is a sex-specific difference. Men showed a more defined dose–response relationship, while women were affected by endocrine regulation, which may weaken exercise-mediated bone protection. In the future, large-scale cross-sex studies should be carried out, combined with dynamic monitoring of hormone levels, to clarify the sex-specific mechanism of CRF affecting bone metabolism.

### Limitations and prospects

The main advantage of this study is that it used advanced testing methods (17), such as isokinetic muscle strength, CPET testing, and QCT bone mineral density measurement. All tests were performed by a professional team at the hospital to ensure accuracy. At the same time, the subjects were all Chinese adults who lived in the same area and did not take drugs that might interfere with muscle, cardiopulmonary endurance, bone metabolism, etc.

The limitations of this study are that no causal inference can be made on the relationship between skeletal muscle strength and CRF and bone density. Secondly, the study lacks control over information such as the subjects’ lifestyle, physical activity level, sex hormones, etc. At the same time, we currently only consider the bone density of the spine, and in the future we will need to add bone density in more parts to verify the correlation. However, we believe that the current research results support and expand the literature on the relationship between skeletal muscle strength and cardiorespiratory endurance and bone health. Many potential covariates were fully adjusted in the model, demonstrating that greater skeletal muscle strength is associated with higher bone density levels in adults aged 30–60 years, and that CRF is significantly correlated with bone density in the overall population. This complements the results of Chinese research on the relationship between cardiorespiratory endurance and bone density. Future studies focusing on longitudinal studies are needed to determine whether strength training and aerobic training can effectively slow the progression of bone loss, and mechanistic studies on the links between skeletal muscle strength and cardiorespiratory endurance and bone density in men and women are needed to fully elucidate the causal relationship.

## Data Availability

The raw data supporting the conclusions of this article will be made available by the authors, without undue reservation.
